# Guided-mode resonant narrowband terahertz filtering by periodic metallic stripe and patch arrays on cyclo-olefin substrates

**DOI:** 10.1038/s41598-018-35515-z

**Published:** 2018-11-22

**Authors:** Antonio Ferraro, Dimitrios C. Zografopoulos, Roberto Caputo, Romeo Beccherelli

**Affiliations:** 1 Consiglio Nazionale delle Ricerche, Istituto per la Microelettronica e Microsistemi (CNR-IMM), Rome, 00133 Italy; 2 Department of Physics, University of Calabria, I-87036 Rende, (CS) Italy

## Abstract

We experimentally and theoretically demonstrate a class of narrowband transmissive filters in the terahertz spectrum. Their operation is based on the excitation of guided-mode resonances in thin films of the low-loss cyclo-olefin polymer Zeonor, upon which aluminum stripe and patch arrays are patterned via standard photolithography. The filters are engineered to operate in low atmospheric loss THz spectral windows, they exhibit very high transmittance and quality factors, compact thickness, and mechanical stability. The dependence of their filtering properties on the geometrical parameters, the substrate thickness and the angle of incidence is investigated, discussing the physical limitations in their performance. This class of filters provides a cost-effective solution for broadband source or channel filtering in view of emerging terahertz wireless communication systems.

## Introduction

Terahertz (THz) frequencies are increasingly capturing the attention of the scientific community, as they represent the less exploited region of the electromagnetic spectrum and they bridge the gap between optics and radiofrequency science and technology. This renewed interest was stimulated by significant improvements in the performance of THz emitters and detectors^1^, which paved the way for novel applications spanning from telecommunications^2,3^, cultural heritage^4^, and life sciences^5^, to security and defense^6^, or industrial non-destructive testing^7^. Therefore, the manipulation of THz wave propagation is of primary importance and much effort has been focused on the development of THz functional components such us polarizers, lenses, amplitude and phase modulators, sensors, and waveplates^8–17^. One class of such fundamental components are transmissive bandpass filters, which select radiation only in a narrow spectrum around a target frequency, a key property in numerous applications, e.g. ultrasensitive sensors^18^, compact spectrometers^19^, telecommunication^20^, or radar science^21^.

A state-of-the-art filter should ideally combine the following features: (i) high peak transmittance (low insertion losses), (ii) narrow linewidth (high quality factor *Q*), (iii) possibility for polarization-independent or tailorable polarization-dependent operation, (iv) low manufacturing cost, and (v) compact overall dimensions. The latter issue can be effectively addressed by employing thin metallic frequency-selective surfaces (FSS) or metasurfaces, which eliminate the need for dielectric multilayers and shrink the component dimensions. A long-established solution for transmitting only a confined set of THz frequencies is based on periodic FSS arrays of cross-shaped apertures, fabricated either as free-standing metallic films or patterned on thin substrates, which however result in rather broad resonances that cannot exceed *Q* values higher than 15^22–30^.

Recently, very high *Q* values have been observed in various metasurface designs with intentional symmetry breaking that excites trapped modes and leads to very sharp Fano resonances, with *Q* values even above 200^31–38^. Nevertheless, such high *Q* values are associated with very low peak transmittance and, in addition, these resonances are embedded in a non-flat background spectral response, which demonstrates very low out-of-band rejection with limited practical relevance. Moreover, since they are based on symmetry breaking, they inherently rule out polarization-independent operation. The latter also stands in the case of asymmetric single split rectangular ring elements, which were demonstrated as high-transmittance/high-*Q* bandpass filters at microwave frequencies^39^. High transmittance and narrow-linewidth THz transmissive filters have been also demonstrated based on interfering metasurfaces, which however result in a bandpass frequency comb and as such do not allow for out-of-band rejection^40^. In a different approach, extensive capabilities were demonstrated by designing terahertz plasmonic filters in the K-space, yet the resulting transmissive filters still show low out-of-band rejection, transmittance, and linewidth^41^.

In this work, all five aforementioned requirements are attained in a new class of THz transmissive selective filtering elements based on planar cyclo-olefin thin films. Contrary to the FSS-based established THz filters that typically transmit the THz wave through subwavelength apertures in a free-standing configuration, the operation of the proposed filters relies on the excitation of guided mode resonances (GMR) stemming from the coupling between waves on periodic structures with modes guided in a dielectric substrate. Although this concept is well-known in the design of optical filters^42–47^ and it can be applied in lower frequency regions of the electromagnetic spectrum, provided a suitable low loss dielectric is available^48,49^, it has been thus far only marginally exploited in the design of THz components^50,51^. Song *et al*. experimentally demonstrated the validity of the approach in the design of bandpass filters at approximately 7 THz, though resulting in both low measured transmittance and quality factors due to the significant losses of the employed polyimide substrate^50^. Recently, we have observed tightly spaced multiple GMR as a secondary effect in the response of FSS THz filters^52,53^. Here, we experimentally demonstrate high-quality GMR transmissive filters able to filter THz radiation in a single operating narrow band, in constrast to the broadband response of standard FSS filters. We focus our design on the two THz low-loss wireless communication windows at 625–725 GHz and 780–910 GHz^3^. These windows are extremely broad, 100 GHz and 130 GHz, respectively. Consequently, dedicated allocation of spectral channels will likely be necessary for different application or different operators. Hence, filters with narrow band and low insertion losses are strategic functional components.

The filters are fabricated by patterning an aluminum layer with a specific design on thin films of the very low-loss cyclo-olefin polymer Zeonor by standard UV photolithography. Low-cost and large-area electronic fabrication processes could be as well employed for ubiquitous deployment in the envisaged short range and indoor THz communication systems^3^. Furthermore, in contrast to free standing metasurfaces, these devices are mechanically stable since they are supported by a polymer substrate. Thanks to the low loss of the substrate, they exhibit peak power transmittance above 85% for *Q* values experimentally measured as high as 70, along with increased out-of-band rejection. In addition, by proper design, both polarization-dependent and independent operation is demonstrated. Finally, a theoretical parametric analysis is presented to provide guidelines on the design of the presented filters and to investigate into their performance limitations. By engineering the design to extreme subwavelength features, we numerically demonstrate that it is possible to squeeze more than half of the incoming THz radiation within a narrow spectral band with a very high quality factor of about 140.

## Terahertz Narrowband Guided-Mode Resonant Transmissive Filters

**Figure 1:**
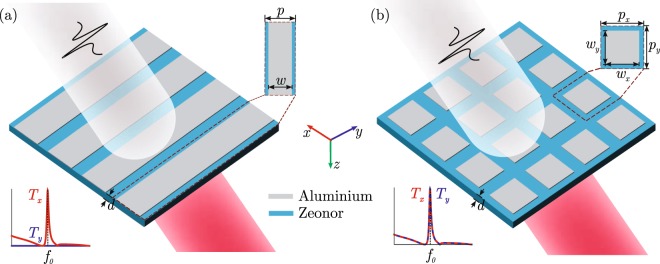
Schematic layout and definition of the geometrical parameters for the investigated GMR filtering elements based on (**a**) stripe and (**b**) square patch aluminum gratings patterned on the cyclo-olefin Zeonor. The thickness of the Al layer is 200 nm.

The layout of the proposed filtering components is schematically depicted in Fig. 1. A periodic configuration of Al stripes or rectangular patches is patterned on a thin film of Zeonor, a cyclo-olefin polymer with outstanding properties, such as high mechanical flexibility, heat resistance, negligible birefringence, and among the lowest absorption and reflection losses at THz frequencies. For the case of normal incidence and THz wave polarized along the grating vector, the diffraction modes of the periodic grating can be coupled to the polymer slab waveguide modes whose propagation constant *β*_eff_ = *n*_eff_*k*_0_ satisfies the phase matching condition

1 $${\beta }_{{\rm{eff}}}=|m|\frac{2\pi }{p},$$

where *n*_eff_ is the effective index of the guided mode, *k*_0_ = 2*π*/*λ*_0_ is the free-space wavenumber, *λ*_0_ the resonant wavelength, *p* the grating pitch, and *m* the diffraction order. In this work, the proposed filters are based on first-order diffraction (*m* = ±1). Equation (1) can be equivalently written as

2 $${n}_{{\rm{eff}}}=|m|\frac{c}{p{f}_{0}},$$

with *f*_0_ being the resonant frequency and *c* the speed of light in vacuum.

The most widespread configuration of GMR filters is based on all-dielectric gratings that operate in reflection, namely they block the transmittance at the GMR frequencies. However, when the grating acts not only as the diffractive element, but also as a highly reflective screen, high transmittance is achieved only in a narrow band around *f*_0_, even for a deeply subwavelength thickness of the filter^45,46^. This property is here exploited for the design of the proposed THz filters, by employing aluminum surfaces patterned on thin Zeonor substrates. The choice of the substrate material is instrumental in providing low intrinsic absorption losses and suitability for processing and handling of the manufactured filters.

Seven different chips, whose geometrical parameters and a brief summary of the key results are reported in Table 1, were designed and fabricated by UV photolithography^15^. A 200-nm layer of aluminum was evaporated on the Zeonor substrates. A film with thickness 1.3 ± 0.1 μm of the photoresist S1813 (Shipley) was spin-coated at 4000 rpm for 60 sec and then cured at a temperature of 115 °C for 120 sec. The photolithography was carried out using a Karl Suss MA150 mask aligner (*λ* = 365 nm, *I* = 60 mW/cm^2^). The samples were immersed in the developer MF319 for 50 sec, rinsed with DI water, dried with nitrogen and cured at 120 °C for 5 min. Finally, the aluminum layer was wet-etched and the residual photoresist was removed with acetone and washed with isopropanol.

**Table 1:** Summary of the geometrical parameters and performance metrics of the fabricated GMR filters.

Chip	*p*_*x*_ (μm)	*p*_*y*_ (μm)	*w*_*x*_ (μm)	*w*_*y*_ (μm)	*d* (μm)	${{\boldsymbol{f}}}_{{\bf{0}}}^{{{\boldsymbol{T}}}_{{\bf{e}}{\bf{i}}{\bf{g}}}}$ (GHz)	${{\boldsymbol{f}}}_{{\bf{0}}}^{{{\boldsymbol{T}}}_{{\bf{f}}{\bf{w}}}}$ (GHz)	${{\boldsymbol{f}}}_{{\bf{0}}}^{{{\boldsymbol{E}}}_{{\bf{T}}{\bf{D}}{\bf{S}}}}$ (GHz)	${{\boldsymbol{Q}}}^{{{\boldsymbol{T}}}_{{\bf{e}}{\bf{i}}{\bf{g}}}}$	${{\boldsymbol{Q}}}^{{{\boldsymbol{T}}}_{{\bf{f}}{\bf{w}}}}$	${{\boldsymbol{Q}}}^{{{\boldsymbol{E}}}_{{\bf{T}}{\bf{D}}{\bf{S}}}}$
#1	390	—	351	—	100	666	667	667	29	29	29
#2	288	—	260	—	100	848	850	849	34	34	31
#3	288	288	260	260	100	848	850	851	34	34	32
#4	390	390	351	351	100	666	667	664	29	29	28
#5	340	340	306	306	100	743	745	748	30	30	30
#6	438	438	394	394	100	605	606	606	28	28	28
#7	288	288	260	260	40	986	985	984	62	62	73

Superscripts (*T*_eig_), (*T*_fw_), and (*E*_TDS_) stand for “Theoretical/eigenfrequency analysis”, “Theoretical/full-wave analysis” and “Experimental TDS measurements” respectively.

Subsequently, they were characterized via terahertz time-domain spectroscopy (THz-TDS) using a Menlo Systems TERA K15 all fiber-coupled spectrometer in transmission mode in nitrogen environment to avoid absorption of THz radiation from water vapor. The spectral characteristics of the sample and reference were calculated via a fast Fourier transformation of the time-domain signals. The spot-size of the collimated beam was ~10 mm in diameter. A terahertz time scan of 800 ps was employed for a spectral resolution of 1.25 GHz. The experimental measurements were directly compared to theoretical finite-element simulations. These were performed via the frequency-domain finite-element method (FEM) implemented in the commercial software COMSOL Multiphysics. In order to calculate the filter transmission spectrum, a unit cell of the periodic structures was simulated by applying Floquet periodic conditions at the lateral lattice borders. The structure was excited by a plane-wave impinging from the top and the power transmittance of the 0-th order diffracted mode was calculated after transmission through the THz-GMR filter. Aluminum was modeled via the impedance boundary condition, assuming Drude permittivity with plasma frequency *ω*_*p*_ = 2.243 × 10^16^ rad/s, and damping rate *γ* = 1.243 × 10^14^ rad/s^54^. The refractive index of Zeonor was *n*_*p*_ = 1.525 − *j*0.0013^55^. Additionally, we performed an eigenfrequency analysis of the investigated structures in order to calculate the guided mode eigenfrequencies in the patterned-Al/Zeonor/air slab waveguide by fixing their propagation constant at *β*_eff_ = 2*π*/*p*, as dictated by the phase matching condition of Eq. (1). The real part of the resulting complex eigenfrequencies *f*_*e*_ gives the guided-mode resonant frequency, while the quality factor is estimated by *Q* = Re{*f*_*e*_}/(2 Im{ *f*_*e*_}). Since material dispersion cannot be incorporated in a straightforward manner, in each studied case the Al permittivity was taken equal to the value given by the Drude model at the resonant frequency observed in the corresponding transmittance spectra. This analysis provides additional evidence that the resonant behaviour of the proposed filters indeed stems from the excitation of guided-modes in the substrate.

In the case of 2D patterns, we have opted for square lattices (*p*_*x*_ = *p*_*y*_ = *p*, *w*_*x*_ = *w*_*y*_ = *w*) in order to induce polarization-independent properties, as it will be demonstrated, working at the equivalent first-order diffraction modes (±1, 0) and (0, ±1). The pitch value for chips #1–4 was selected such that the resonant frequencies lie in the center of the low-loss atmospheric attenuation windows envisaged for next-generation THz wireless communications, i.e. 625–725 GHz and 780–910 GHz^3^. The rest of the chips (#5–7) were designed so as to provide a parametric study of their filtering properties. In all cases the fill factor of the metal layer, here defined as *F* = *w*/*p*, is fixed at 0.9, which provides a good compromise between high peak transmittance, high quality factors, and increased out-of-band rejection. Larger fill factors would provide narrower band operation at the cost of reduced transmission as discussed later on.

**Figure 2:**
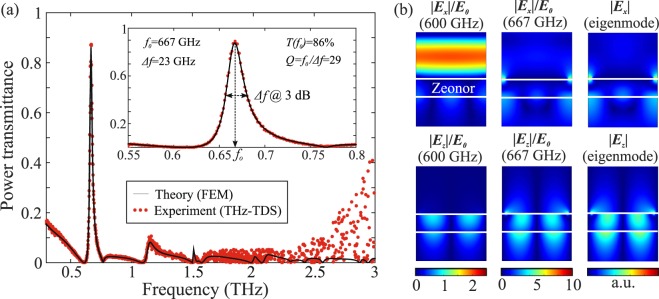
(**a**) Theoretically calculated and experimentally measured transmittance in the 0.3–3 THz range for an Al-stripe GMR filter with *p* = 390 μm, *w* = 351 μm, and *d* = 100 μm (chip #1) and relevant performance metrics. The inset shows a zoom around the resonant frequency of 667 GHz. The THz-TDS data above 2.5 THz show some scattering owing to the decreasing signal-to-noise ratio of the instrument. (**b**) Relative electric field components calculated via full-wave simulation off (600 GHz) and on (667 GHz) resonance. Note the different scales in the two cases for the field enhancement. The field profiles on resonance match those of the guided mode calculated at 666 GHz through eigenfrequency analysis.

Figure 2 reports the power transmittance simulated and experimentally measured for chip #1. The filter exhibits very high transmittance (*T* = 86%) and a 3-dB linewidth Δ*f* = 23 GHz calculated at the full-width half-maximum (FWHM) around the resonant frequency *f*_0_ = 667 GHz, which corresponds to a quality factor *Q* = *f*_0_/Δ*f* = 29. We have numerically calculated that the insertion losses of 14% (0.65 dB) at the resonant frequency stem from the very low but finite absorption in Zeonor (5%) and Al (2%), and reflection from the device (7%). This was verified by simulating the filter transmittance spectrum first by considering lossless conditions for Al (zero damping in the Drude model) and Zeonor (*n*_*p*_ = 1.525), which led to *T* = 93% at resonance. The inclusion of Al losses reduced the transmittance to 91% and the inclusion of both Al and Zeonor losses led to the calculated value of *T* = 86%, which was experimentally measured, as shown in Fig. 2. Furthermore, the out-of-band transmittance of the filter stays below 10% in the whole spectral range between 0.5 and 3 THz. These results correspond to an impinging THz wave that is polarized perpendicularly to the Al stripes, i.e. along the *x*-axis. For the cross-polarization the Al grating acts as a nearly ideal mirror. Such polarization-sensitive behavior was investigated in the case of chip #2, which is also based on Al-stripe lattice, and designed to have a resonant frequency at 850 GHz.

In order to elucidate more on the physics behind the observed resonance in the transmittance spectrum of the device, we have calculated the profiles of the electric field components via FEM full-wave simulations off- and on-resonance, at the frequency of 600 and 667 GHz, respectively. The results are shown in Fig. 2(b) and they are expressed normalized to the amplitude of the impinging x-polarized planewave. At 600 GHz almost 100% of the incoming power is reflected, creating the expected standing wave pattern in the half-space above the device, the reflected wave being also x-polarized. On-resonance, both components of the electric field are enhanced, not only at the tips of the Al stripes^56^, but also in the Zeonor substrate owing to the excitation of the resonant guide mode. This profile corresponds to the first-order TM-polarized mode guided in a Al/Zeonor/air slab waveguide, as discussed in ref.^53^. To further corroborate that the resonance indeed stems from coupling to guided modes, we have plotted in Fig. 2 the profiles of the electric field components of the eigenmode calculated at 666 GHz via an eigenfrequency analysis. The profile of the electric field excited by the planewave impinging on the filter at the resonant frequency corresponds to the calculated guided mode in the substrate.

**Figure 3:**
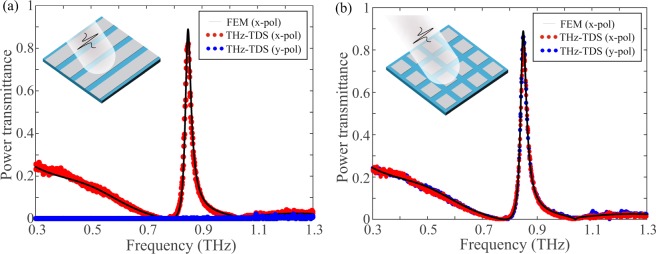
Transmittance for (**a**) stripe (chip #2) and (**b**) patch array GMR filtering components (chip #3) with *p* = *p*_*x*_ = *p*_*y*_ = 288 μm, *w* = *w*_*x*_ = *w*_*y*_ = 260 μm, *d* = 100 μm. Polarization-dependent and polarization-independent filtering is, respectively, achieved.

**Figure 4:**
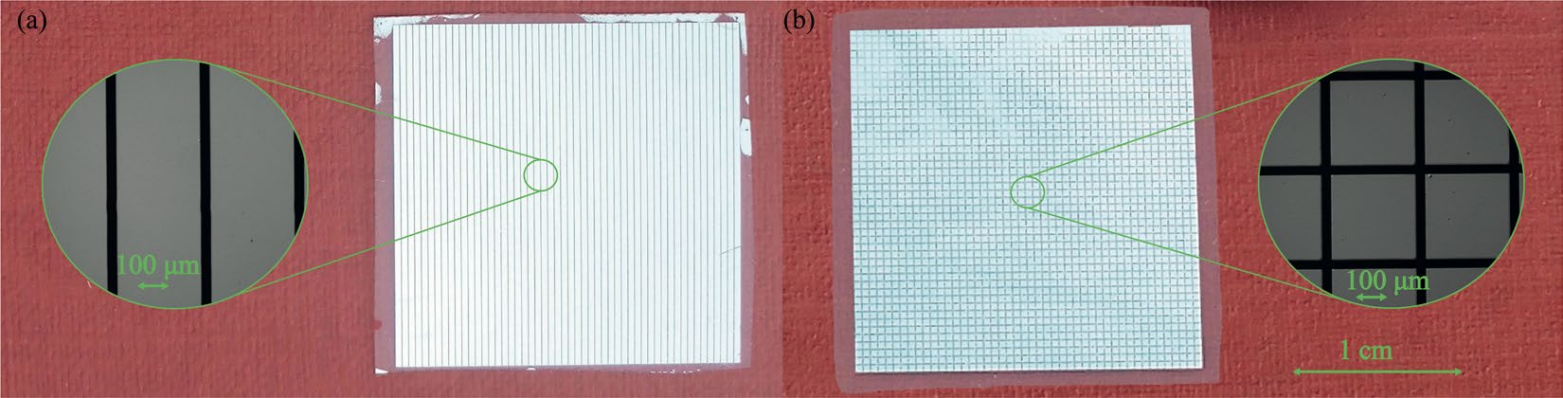
Photographs of (**a**) chip #2 and (**b**) chip #3 and corresponding zoomed micrographs taken under microscope in reflection mode, showing the Al periodic stripe and patch arrays defined by photolithography on the Zeonor substrate.

**Figure 5:**
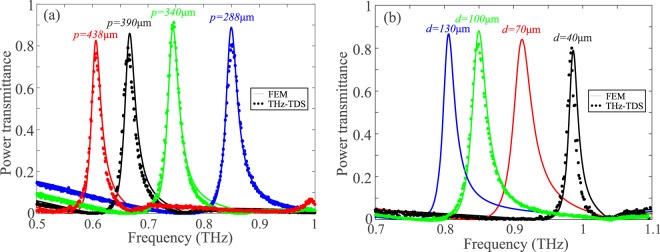
(**a**) Theoretically calculated and experimentally measured transmittance of 2D GMR filters for different values of the lattice pitch (chips #3–6). The fill factor is kept constant at 90% and *d* = 100 μm. (**b**) Theoretically calculated and experimentally measured transmittance of 2D GMR filters for *p*_*x*_ = *p*_*y*_ = 288 μm, *w*_*x*_ = *w*_*y*_ = 260 μm and different values of the Zeonor substrate thickness. The fabricated chips (chips #3 and #7) correspond to the available Zeonor film thicknesses of *d* = 40 and 100 μm.

Figure 3 shows the polarization-dependent transmittance of chip #2, compared to that of chip #3, which comprises a square lattice of Al patches with the same pitch and patch width. It is evident that the proposed stripe-based GMR filters transmit only one polarization, while their patch counterparts exhibit polarization-independent performance. The latter cannot be achieved in high-*Q* THz filters induced by symmetry breaking, which do not exhibit the *C*_4_ symmetry of the square lattice GMR filters, as it can be visualized in Fig. 4(b) that shows the photo and micrograph of the patterned surface of chip #3. The corresponding photos for chip #2, characterized by the array of Al stripes, are shown in Fig. 4(a). The exact position of the resonant frequencies depends mainly on the lattice pitch and the slab mode effective index that, for a fixed polymer material refractive index, is determined by the substrate thickness. For a given substrate film thickness, the resonant frequency can be readily adjusted by properly tailoring the lattice pitch, as demonstrated in Fig. 5(a), which reports the transmittance of chips #3–6 that correspond to four different pitch values. In all cases the peak transmittance stays high, above 80%. The filter quality factor does not significantly vary and stays around *Q* = 30. Excellent agreement between THz-TDS measurements and FEM simulations is observed. In addition, Fig. 5(b) investigates the effect of the substrate thickness *d* for a fixed pitch *p* = 288 μm. Samples were fabricated for two of the four investigated cases, namely those with thicknesses *d* = 40 and 100 μm. This selection was solely limited by the current availability of Zeonor films. In principle, for any substrate thickness the operation frequency can be tuned by properly engineering the filter geometry. As the film thickness shrinks, the slab modal index drops and thus the resonant frequency is shifted toward the limit *f*_max_ = *c*/*p*, which is around 1.04 THz, also identifiable by considering the zero-transmittance point associated with Wood’s anomaly^57^. For all the investigated films thicknesses, the power transmittance remains very high, between 80 and 90%. In particular, for *d* = 40 μm (chip #7), this very high transmittance level is accompanied by a very high *Q*-factor value of 73.

**Figure 6:**
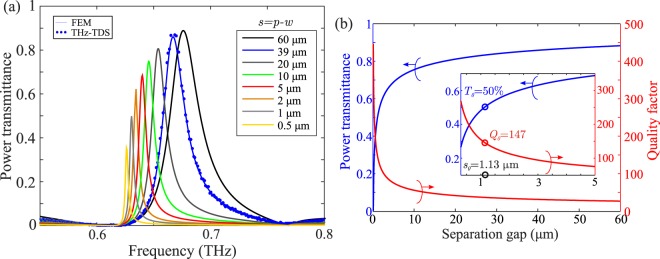
(**a**) Transmittance of a 2D GMR filter with *p* = 390 μm and *d* = 100 μm, as a function of the gap *s* = *p* − *w* in the Al patch lattice. (**b**) Dependence of the filter transmittance and quality factor on the gap value. For *s* = 1.13 μm (*F* = 0.997), a *Q* = 147 is obtained for 3 dB losses.

In all cases examined so far, the fill factor of the metallic lattice was kept equal to 90%. By varying the fill factor, or equivalently the separation gap *s* = *p* − *w* between adjacent Al patches, the effect on the filtering characteristics of chip #4 is shown in Fig. 6. As the gap shrinks, the resonance linewidth becomes narrower at the expense of reduced transmittance. However, even in the extreme case of gap values below 1 μm, i.e. almost 500 times smaller than the free-space wavelength, a significant amount of THz power still crosses the filter. This tight squeezing of electromagnetic radiation in extremely subwavelength slits and volumes has also been observed in non-diffracting gratings^56^. Figure 6(b) provides a design rule for the selection of the gap value, in accordance with the target performance characteristics. It is remarked that for *s* = 1.13 μm (*F* = 0.997), which can still be fabricated with standard UV photolithography without resorting to nanofabrication techniques, a quality factor as high as 147 is obtained for insertion losses of 3 dB. The combination of such high quality factors for single bandpass 50% filtering in transmission is a remarkable achievement in the field of THz technology, thanks to the careful electromagnetic design and the intrinsic low-loss properties of the substrate. The optimal selection of the fill factor value depends on the specific requirements of the target application.

It is known that when a plane-wave impinges obliquely on a GMR filter the resonances for each diffracted mode split and follow two separate spectral branches as the angle of incidence *θ* of the probe radiation increases^43,53^. In particular, the phase-matching condition of Eq. (1) for the case of oblique incidence takes the form

3 $${\beta }_{{\rm{eff}}}=|{k}_{0}\,\sin (\theta )-m\frac{2\pi }{p}|,$$

which gives two different solutions corresponding to *m* = 1 and *m* = −1. This is contrary to the case of normal incidence where the two modes are degenerate and as a consequence only one peak is observed in the transmittance spectrum. The effective indices of the two excited modes are given by

4 $${n}_{{\rm{eff}}}^{-}=\frac{c}{p{f}_{0}^{-}}+\,\sin (\theta ),\,{n}_{{\rm{eff}}}^{+}=\frac{c}{p{f}_{0}^{+}}-\,\sin (\theta ),$$

where the resonant frequencies ${f}_{0}^{-}$ (${f}_{0}^{+}$) are lower (higher) than *f*_0_(*θ* = 0°).

**Figure 7:**
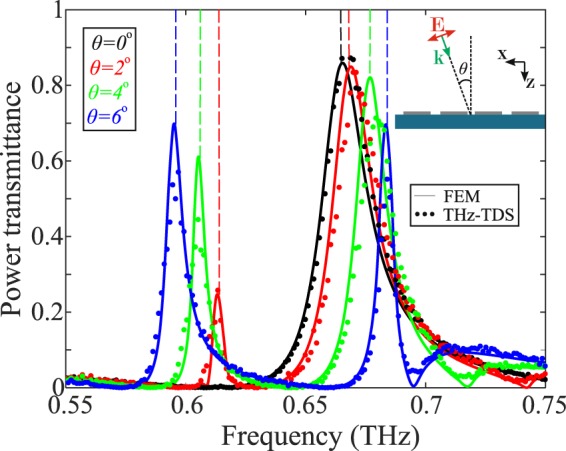
Theoretically calculated, via FEM full-wave simulations, and experimentally measured transmittance of chip #1 as a function of the angle of incidence of the probe radiation planewave. The dashed lines indicate the resonant frequencies as calculated through the eigenfrequency analysis.

We characterized chip #1 for *θ* = 0°, 2°, 4°, and 6° and plotted the obtained experimental results in Fig. 7. The resonance splitting is evident and in good agreement with theoretical modeling. The dashed lines indicate the frequencies corresponding to the eigenfrequency analysis problem, showing agreement better than 1.5 GHz, consistent with the results presented in Table 1. The tweaking of the angle of incidence using a rotation stage provides a means to dynamically tune the filter resonant frequency in a broad range and/or to achieve dual-band operation. For instance, the single resonance observed at normal incidence degenerates for *θ* = 6° into two resonances, with nearly identical transmission (*T* = 70%), centered at 595 GHz and 683.5 GHz. Interestingly, these resonances are characterized by much higher quality factors (70 and 100, respectively) compared to the single one observed at normal incidence. This may find applications in filtering the transmit and receive communication channels using a single filter.

## Conclusions

To sum up, a new class of THz filtering elements based on GMR in Al gratings supported by thin films of the low-loss cyclo-olefin polymer Zeonor is investigated, both theoretically and experimentally. The proposed filters combine a series of performance qualities, i.e. very high transmittance, narrow linewidth, increased out-of-band rejection, compact size, mechanical stability, and low manufacturing cost, which are unprecedented in the field of THz filter technology. Both polarization-selective and polarization-independent response is demonstrated by toggling between the dimensionality of the patterned Al lattice. Although part of the presented filters is specifically engineered for use at the low-absorption THz atmospheric windows, the general design procedure and rules are also discussed. Finally, the GMR-splitting under oblique illumination is also examined as a means to dynamically tailor the filter resonant frequency.
